# Genistein inhibits the replication of enterovirus A71

**DOI:** 10.3389/fphar.2026.1787050

**Published:** 2026-04-17

**Authors:** Wenbo Huo, Yahong Zhang, Cong Wang, Changrui Huang, Xinhe Zhou, Siyu Liu, Hongying Bai, Xiaoyan Yu, Jinghua Yu

**Affiliations:** 1 Department of Experimental Pharmacology and Toxicology, School of Pharmaceutical Science, Jilin University, Changchun, Jilin, China; 2 Infectious Diseases and Pathogen Biology Center, The First Hospital of Jilin University, Jilin University, Changchun, Jilin, China; 3 Institute of Virology and AIDS Research, The First Hospital of Jilin University, Jilin University, Changchun, Jilin, China; 4 State Key Laboratory of Antiviral Drugs, Henan University, Kaifeng, Hennan, China

**Keywords:** autophagy, EV71, G2/M arrest, genistein, viral replication

## Abstract

**Background:**

Genistein, an isoflavone abundant in soybeans and other legumes, has shown antiviral activity against several viruses. Its effects on enteroviruses, particularly EV71—the causative agent of hand, foot, and mouth disease (HFMD)—remain incompletely understood.

**Methods:**

*In vitro* assays were used to evaluate replication inhibition of EV71, assess mechanistic effects, and determine the compound’s broad-spectrum activity against other enteroviruses (EV68 and CA6). *In vivo* experiments in neonatal mice were conducted to further elucidate the role of genistein in EV71 replication.

**Results:**

Genistein inhibited EV71 replication, as evidenced by reduced viral genome copies, decreased viral protein expression, and lower virion numbers. The compound also reduced replication of EV68 and CA6. Mechanistically, autophagy inhibition contributed to the suppression of EV71 replication, and restoring autophagy attenuated this effect. Additionally, genistein caused a G2/M cell cycle arrest, contributing to impaired EV71 replication. In neonatal mice, genistein conferred protection against EV71-associated disease.

**Conclusion:**

The compound effectively suppresses EV71 replication and mitigates EV71-induced pathology, with concurrent activity against EV68 and CA6, and involves autophagy modulation and cell cycle disruption as part of its antiviral mechanism.

## 1 Introduction

Human enteroviruses are non-encapsulated, single-stranded, positive RNA viruses that belong to the genus *Enterovirus* and the family *Picornaviridae* (Hu et al., 2020). Enterovirus A71 (EV71) is a type of enterovirus that causes typical hand, foot, and mouth disease (HFMD), which continues to pose a significant public health challenge. Existing EV71 vaccines provide limited cross-protection (Ng et al., 2015; Jin et al., 2016), and there are no targeted therapeutic drugs available in clinics, highlighting the urgent need for antiviral treatments to combat enteroviral infections (Pei et al., 2022).

**FIGURE 1:**
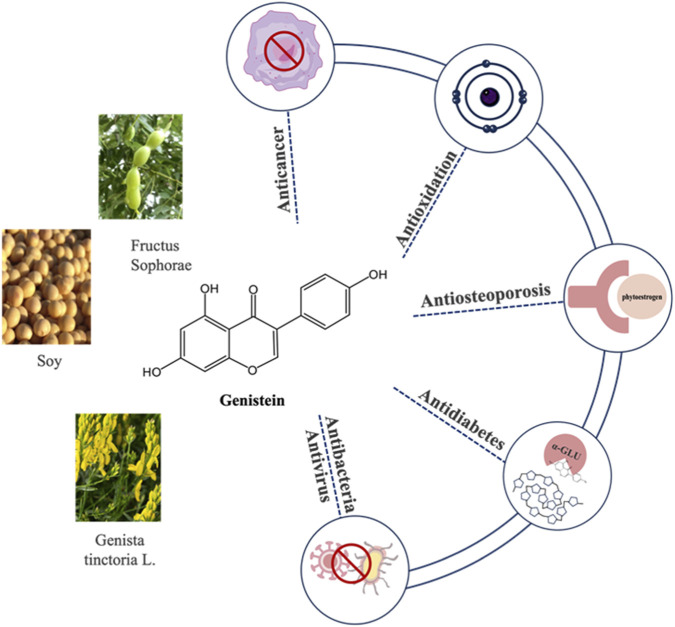
The introduction of genistein.

Genistein, a prevalent isoflavone, was first identified in *Genista tinctoria* L., from which it derives its name (Figure 1). Genistein is widely distributed in leguminous plant foods, as well as in seeds, fruits, and vegetables such as alfalfa and clover sprouts, broccoli, cauliflower, sunflower, barley meal, caraway, and clover seeds. Genistein primarily occurs in nature as the β-glycoside forms of genistein, which are not bioavailable. Upon ingestion, enzymes β-glycosidases in the small intestinal brush border membrane remove the glycoside group, allowing genistein to be readily absorbed and become bioactive (Křížová et al., 2019). Due to the long history and widespread consumption of soybeans in Asian cuisine, coupled with their generally recognized safety, the U.S. Food and Drug Administration approved health claim labeling for soy-based foods in 1999, asserting their role in reducing the risk of coronary heart disease (Vincent and Fitzpatrick, 2000; Rimbach et al., 2008). Since 1998, a total of 67 Phase I-III clinical trials have been registered with the National Institutes of Health to evaluate the biological benefits of genistein and its derivatives (Middleton et al., 2000; Rizzo and Baroni, 2018; Mathew and Vazhappilly, 2020) (https://clinicaltrials.gov/). Various studies have reported that genistein possesses a wide range of biological and pharmacological properties, including antioxidant, antiangiogenic, anti-inflammatory, anti-tumor, antibacterial, antiviral, and anthelmintic effects, as well as pharmacological activities related to diabetes and lipid metabolism (Middleton et al., 2000; Martin et al., 2007; Andres et al., 2009; Fuloria et al., 2022).

Autophagy is the process by which cells degrade and recycle proteins and organelles to maintain intracellular homeostasis (Deretic et al., 2013). In the course of viral replication, autophagy plays multiple roles in viral infection, functioning either to support or inhibit the virus. On one hand, the host cell rapidly initiates autophagy to degrade virus particles or viral components upon infection and works together with the antiviral interferon response to suppress viral replication. On the other hand, viruses induce autophagy and then hijack autophagosomes as replication sites or exploit the secretory autophagy pathway to promote the maturation and release of virus particles, thereby enhancing replication and transmission efficiency (Choi et al., 2018). Genistein has been reported to decrease autophagy in some studies (Mohan et al., 2013; Nazim and Park, 2015; Wu et al., 2023; Wu et al., 2024) while increase it in others (Lee et al., 2016; Wang et al., 2018; Pierzynowska et al., 2019; Zhang et al., 2019; Pierzynowska et al., 2024). Therefore, the regulation of genistein on autophagy should be investigated and confirmed.

Viruses utilize various strategies to disrupt cell cycle checkpoint controls and manipulate cell proliferation pathways. Several viruses produce proteins that target key cell cycle regulators to create cellular conditions favorable for viral replication (Nascimento et al., 2012; Bagga and Bouchard, 2014). EV71 replication causes cell cycle arrest in the S phase, and conversely, cells arrested in the S phase provide conditions that support EV71 production, whereas G2/M phase arrest inhibits viral replication (Yu et al., 2015).

In this study, we investigated the anti-EV71 mechanism of genistein and confirmed for the first time that genistein regulates intracellular homeostasis—particularly by inhibiting autophagy and inducing cell cycle arrest—to suppress EV71 replication.

## 2 Materials and methods

### 2.1 Ethic statements

Animal experiments were carried out at the Norman Bethune Medical College Animal Experiment Center of Jilin University (Changchun, China) in accordance with Ethics Committee’s regulations after approval.

### 2.2 Cells

Human rhabdomyosarcoma RD cells (CCL-136) and human cervical cancer HeLa cells (CCL-2TM) were purchased from the American Type Culture Collection (ATCC, Manassas, VA, USA). RD and HeLa cells were cultured in Dulbecco’s modified Eagle’s medium (DMEM, 10–013-CVRC, Corning, New York, United States) with 10% fetal bovine serum (FBS, 086–110, Wisent Inc., St-Bruno, Quebec, Canada) and Penicillin-Streptomycin solution (100 U/mL Penicillin and 100 μg/mL Streptomycin). They were cultured at 37 °C in a humidified incubator with 5% CO2.

### 2.3 Viruses

EV71 (Changchun 077 strain) was purified at the first hospital of Jilin University. EV68 (US/KY/14–18953 strain, VR-1825D) were purchased from ATCC. CA6 (98 strain) was obtained from the Jilin Provincial Center for Diseases Control and Prevention. They were preserved at −80 °C in the First Hospital of Jilin University.

### 2.4 Compounds and reagents

Genistein (C15H10O5) with purities higher than 98% determined by HPLC (B21039) was purchased from Yuanye Biotechnology Co., Ltd. (Shanghai, China). It was stored at −20 °C in a powder state or dissolved in dimethyl sulfoxide (DMSO). Bafilomycin A1 (GC17597), Z-VAD-FMK (GC12861), MG132 (GC10383), and 3-methyladenine (3-MA, GC10710) were purchased from GlpBio (Montclair, CA, USA). All of them were dissolved in DMSO at −20 °C.

### 2.5 Cytotoxicity assay

To observe the effect of genistein on cell growth, cytotoxicity assay was carried out. When RD or HeLa cells reached approximately 80% confluence in a 96-well plate, 0, 4, 8, 16, 32, 64, 128, and 256 μM of genistein (prepared in DMEM with 10% FBS) and vehicle control (10% DMEM with DMSO, the genistein solvent) were added and incubated for 24 h. After treatment, 10 µL of Cell Counting Kit-8 (CCK8; GK10001, GlpBio) was added to each well (100 µL final volume) and incubated at 37 °C for 2 h. Absorbance was measured at 450 nm using a microplate reader (Bio-Rad Laboratories, Hercules, CA, USA). Cell viability was calculated as:

$$\text{Cell Viability }\left(\%\right)=\left[\left(\text{As}-\text{Ab}\right)\;/\;\left(\text{Ac}-\text{Ab}\right)\right]\times 100$$

where: As = Absorbance of sample well (cells + medium + genistein + CCK8); Ac = Absorbance of control well (cells + medium + CCK8); Ab = Absorbance of blank well (medium + CCK8).

### 2.6 Western blot and antibodies

To analyze the expression of viral protein and host protein, Western blot was performed. After collection, cells were washed once with 500 µL of PBS (phosphate-buffered saline) and then lysed using Lysis Buffer (P0013, Beyotime Biotechnology, Shanghai, China). The lysate was mixed with protein loading buffer. Proteins were separated by SDS-PAGE and transferred from the gel onto a 0.22 µm PVDF membrane using a semi-dry transfer apparatus. The membrane was incubated with the appropriate primary antibody, followed by an HRP-conjugated secondary antibody. Electrochemiluminescence (MA0186, Meilun Biotechnology, Dalian, China) was used to detect protein levels. Alpha Tubulin antibody (80762-1-RR), GAPDH monoclonal antibody (60004-1-Ig), IkB Alpha polyclonal antibody (10268-1-AP), caspase3 polyclonal antibody (19677-1-AP), P62/SQSTM1 polyclonal antibody (18420-1-AP), LC3 polyclonal antibody (14600-1-AP), CD107a/LAMP1 polyclonal antibody (21997-1-AP), CDK4 polyclonal antibody (11026-1-AP), cyclinB1 polyclonal antibody (55004-1-AP), CDK1 polyclonal antibody (19532-1-AP), cyclinE1 polyclonal antibody (11554-1-AP), CDK2 polyclonal antibody (10122-1-AP), and cyclinD1 monoclonal antibody (60186-1-Ig) were purchased from Proteintech (Wuhan, China). EV71 VP1 monoclonal antibody (GTX132338), EV68 VP1 polyclonal antibody (GTX132313), and CA6 VP1 polyclonal antibody (GTX87102) were purchased from GeneTex (Irvine, CA, USA). Quantitation of Western blotting was measured by ImageJ software (Version 2.3.3/1.53q).

### 2.7 RNA extraction, reverse transcription PCR, and real-time PCR

To detect the viral mRNA level, real-time PCR was done. RD or HeLa cells cultured in 6-well plates and infected mock and virus. At 24 h post-infection, cells were washed once with 1× PBS, Total RNA was directly lysed in the well by adding 1 mL of TransZol and incubating at room temperature for 5 min. The resulting lysate was transferred to a nuclease-free 1.5 mL microcentrifuge tube. Total RNA was purified from the lysate using the TransZol Up Plus RNA Kit (ER501-01, TransGen Biotech, Beijing, China) according to the manufacturer’s instructions for column-based purification. RNA concentration and purity were measured spectrophotometrically. Purified RNA was reverse transcribed into first-strand cDNA using the TransScript® One-Step gDNA Removal and cDNA Synthesis SuperMix (AT341-02, TransGen Biotech) following the manufacturer’s protocol. Quantitative PCR was conducted using the TransStart® Tip Green qPCR SuperMix (AQ601-02, TransGen Biotech) on a real-time PCR detection system. The reactions contained cDNA template, gene-specific primers, and qPCR master mix in a final volume of 20 µL. Thermocycling conditions were as per kit recommendations (95 °C for 30 s, followed by 40 cycles of 95 °C for 5 s, 60 °C for 30 s).

The following primer pairs were used:

GAPDH  Forward: 5′- GCA​AAT​TCC​ATG​GCA​CCG​T -3';
      Reverse: 5′- TCG​CCC​CAC​TTG​ATT​TTG​G -3′
EV71-VP1 Forward: 5′- AGC​ACC​CAC​AGG​CCA​GAA​CAC​AC -3';
      Reverse: 5′- ATC​CCG​CCC​TAC​TGA​AGA​AAC​TA -3′
EV68-VP1 Forward: 5′- GCC​CTT​ACT​CCA​GAA​AAA​CA -3';
     Reverse: 5′- CAA​AAC​CAT​CAT​AGA​AAA​CT -3′
CA6-VP1 Forward: 5′- AAT​GAG​GCG​AGT​GTG​GAA​C -3';
     Reverse: 5′- AGG​TTG​GAC​ACA​AAA​GTG​AAC​T -3′

### 2.8 Determination of the virus titer by plaque assay

To detect the virion number, the virus titer by plaque assay was carried out. The supernatant from EV71-infected cells was collected and diluted. RD cells, seeded in 12-well plates at 90% confluency, were infected with the diluted supernatant for 2 h. After infection, the inoculum was replaced with an overlay medium containing 1.6% low-melting-point agarose and 2% FBS in maintenance medium. Following 30 h of incubation, the cells were fixed with 4% paraformaldehyde and stained with 0.1% crystal violet. The number of plaques per well was counted and multiplied by the dilution factor to calculate the viral titer (in PFU/mL).

### 2.9 Determination of the virus titer by TCID50

To detect the virion number, the virus titer by TCID50 was carried out. Collect the supernatant and intracellular virus at the designated time, centrifuge at 10,000 g at 4 °C for 5 min. Dilute with DMEM at a 10 fold gradient and add it to a 96 well plate. After 36 h, count the number of cells with cytopathic effect at each concentration, and calculate TCID50/mL based on the number of cells with cytopathic effect.

### 2.10 Plasmids and transfection reagent

To observe the autophagic intensity, the plasmid of PEGFP-C1-LC3B was transfected. The plasmid was kindly provided by He Hongxuan from the Institute of Zoology, Chinese Academy of Sciences. The transfection reagent used is Yisheng’s liposome transfection reagent (40802ES03, Shanghai, China). Transfection is conducted under serum-free conditions at a plasmid to transfection reagent ratio of 1:2.5. After 6 h, the medium is replaced with 10% DMEM.

### 2.11 Flow cytometry analysis

To observe the cell cycle distribution, flow cytometry was performed after propidium iodide staining. HeLa or RD cells grown in a 6-well plate were washed once with PBS. The cells were detached by trypsinization. The resulting cell suspension was centrifuged at 1,000 rpm for 10 min to pellet the cells. HeLa or RD cells were fixed with ice-cold 70% ethanol and stored at −20 °C for 24 h. After 24 h, the fixation solution was removed by centrifugation. The cells were resuspended in a propidium iodide staining solution (50 μg/mL) and incubated in the dark at 4 °C for 2 h DNA content quantification was performed using a BD FACSCalibur flow cytometer. Cell cycle phase distribution was determined by ModFit LT software (v5.0, Verity Software House, Topsham, ME, USA).

### 2.12 Mice experiment

To analyze the protection of genistein on host, *in vivo* experiment was done in neonatal mice. Pregnant ICR mice were obtained from Yisi Experimental Animal Technology Co., Ltd. (Changchun, China). Within 24 h after birth, each pup received an intracerebroventricular injection of 20 μL EV71 (10^7.0^ CCID50/mL) into the bilateral lateral ventricles, while control pups received an intracerebroventricular injection of 20 μL DMEM. Starting the day after virus inoculation (Day 1 post-infection), mice were given intraperitoneal injections of genistein (10 mg/kg or 50 mg/kg). Control mice received intraperitoneal injections of saline containing 8% DMSO (vehicle control) at an equivalent volume. Drug or vehicle administration was performed once daily for 7 consecutive days. Body weight, survival rate, clinical scores and histopathological analysis were measured and recorded daily throughout the experiment within 14 days.

### 2.13 Quantification and statistical analyses

All data were presented as means ± SD and analyzed using GraphPad Prism software (Version 10.2.0). One way ANOVA analyses were used for comparisons of data with greater than two groups. T-test was used for comparisons of data with two groups. Sample sizes were indicated in the figure legends. Data sets were considered significantly different if the P value was less than 0.05.

## 3 Results

### 3.1 Genistein inhibits viral replication

**FIGURE 2:**
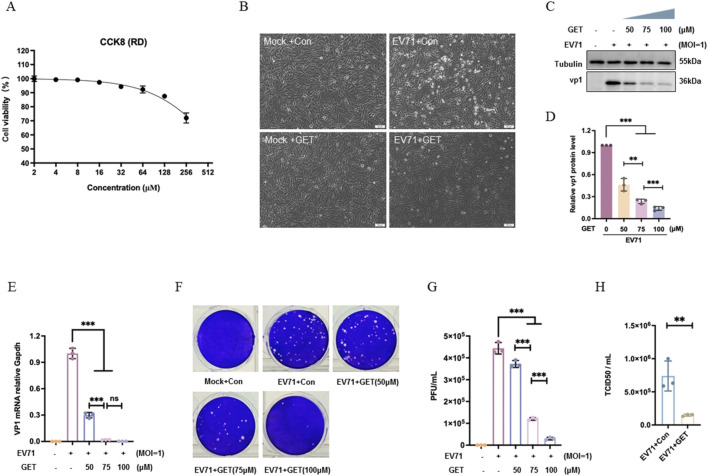
Effects of the genistein on EV71 replication in RD cells. **(A)** Cytotoxicity of genistein on RD cells. RD cells were treated with serially diluted genistein for 24 h, after which the cell viabilities were measured by cell counting kit-8 assay (CCK8). N = 3 replicates in each concentration. **(B)** RD cells were infected with EV71 at a MOI of 1 for 2 h. At 2 h post-infection, cells were treated with 10% DMEM or genistein (GET, 75 μM) for 22 h. Morphology was observed using inverted microscope. N = 3 independent experiments. **(C)** At 2 h post-infection, cells were treated with genistein (0, 50, 75, 100 μM) for 22 h. Western blot analysis of VP1 was performed. Tubulin was used as a loading control. **(D)** Corresponding to **(C)** the gray value ratio of the VP1 protein band to the Tubulin protein band was shown, data were presented as the mean ± SD (N = 3 independent experiments); statistical analysis was performed using one-way ANOVA test with Tukey’s multiple comparisons test. (***P < 0.001; **P < 0.01; *P < 0.05). **(E)** At 2 h post-infection, cells were treated with genistein (0, 50, 75, 100 μM) for 22 h. Relative mRNA levels of viral genome were detected using primers targeting VP1. GAPDH was used as a housekeeping gene. Data were presented as the mean ± SD (N = 3 independent experiments); statistical analysis was performed by one-way ANOVA test with Tukey’s multiple comparisons test (***P < 0.001; ns: no significant difference). **(F)** At 2 h post-infection, cells were treated with genistein (0, 50, 75, 100 μM) for 22 h. Virus from the supernatant was collected for plaque assay analysis. Plaques in RD cells were counted after a 5000-fold dilution of the supernatant. **(G)** Corresponding to **(F)**, data were presented as the mean ± SD (N = 3 independent experiments); statistical analysis was performed by one-way ANOVA test with Tukey’s multiple comparisons test. (**P < 0.01; ***P < 0.001). **(H)** At 2 h post-infection, cells were treated with genistein (75 μM) for 22 h. Intracellular and supernatant progeny virions were titrated using RD cells to determine TCID50/ml. Data were presented as the mean ± SD (N = 3 independent experiments); statistical analysis was performed using T-test (**P < 0.01).

Following treatment with serial dilutions, cell viability in RD was higher than 90% at genistein concentration below 75 μM (Figure 2A), and 75 μM of genistein was used throughout this study. RD cells were infected with EV71 at a multiplicity of infection (MOI) of 1 for 2 h. After 2 h, the cells were washed once with PBS and then treated with 75 μM genistein. At 24 h post-infection, the cytopathic effect was recorded. EV71 infection caused cytopathic effects in RD cells, characterized by rounded and detached cells, whereas treatment with 75 μM genistein inhibited this EV71-induced cytopathic effect (Figure 2B). Additionally, genistein at concentrations of 50 μM, 75 μM, and 100 μM significantly inhibited viral protein expression in a dose-dependent manner (Figures 2C,D, ***P < 0.001). Treatment with 75 μM genistein reduced viral genomic levels compared to the control treatment (Figure 2E, ***P < 0.001). Genistein also inhibited plaque formation (Figures 2F,G, ***P < 0.001) and decreased TCID50 (Figure 2H, **P < 0.01), indicating that genistein decreased virion number. Therefore, genistein inhibited viral replication in RD cells.

**FIGURE 3:**
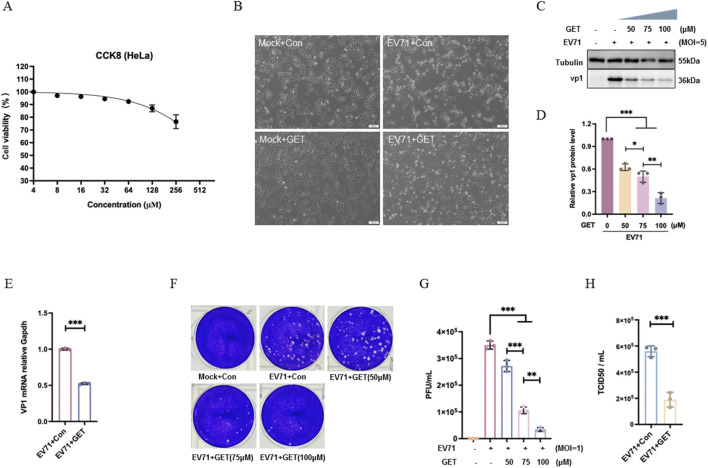
Effect of the genistein on EV71 replication in HeLa cell. **(A)** Cytotoxicity of genistein on HeLa cells. HeLa cells were treated with serially diluted genistein for 24 h, after which the cell viabilities were measured by cell counting kit-8 assay (CCK8). N = 3 replicates in each concentration. **(B)** HeLa cells were infected with EV71 at a MOI of 5 for 2 h. At 2 h post-infection, cells were treated with 10% DMEM or genistein (GET, 75 μM) for 22 h. Morphology was recorded using an inverted microscope. N = 3 independent experiments. **(C)** At 2 h post-infection, cells were treated with genistein (0, 50, 75, 100 μM) for 22 h. Western blot analysis of VP1 was performed. Tubulin was used a loading control. **(D)** Corresponding to **(C)** the gray value ratio of the VP1 protein band to the Tubulin protein band was shown. Data were presented as the mean ± SD (N = 3 independent experiments); statistical analysis was performed by one-way ANOVA test with Tukey’s multiple comparisons test. (***P < 0.001). **(E)** At 2 h post-infection, cells were treated with genistein (75 μM) for 22 h. Relative mRNA levels of viral genome were measured using VP1 primers. GAPDH was used as a housekeeping gene. Data were presented as the mean ± SD (N = 3 independent experiments); statistical analysis was performed using T-test. (***P < 0.001). **(F)** At 2 h post-infection, cells were treated with genistein (0, 50, 75, 100 μM) for 22 h. Virus from the supernatant was collected for plaque assay analysis. Plaques in RD cells were recorded after diluting the supernatant 5000-fold. **(G)** Corresponding to **(F)** data were presented as the mean ± SD (N = 3 independent experiments); statistical analysis was performed using one-way ANOVA test with Tukey’s multiple comparisons test. (**P < 0.01, ***P < 0.001). **(H)** At 2 h post-infection, cells were treated with genistein (75 μM) for 22 h. Intracellular and supernatant progeny virions were titrated using RD cells to determine TCID50/ml. Data were presented as the mean ± SD (N = 3 independent experiments); statistical analysis was performed using a T-test (***P < 0.001).

To verify that genistein’s antiviral effect is not limited to RD cells, HeLa cells were used to evaluate its antiviral activity. Following treatment with serial dilutions, cell viability in HeLa cells was higher than 90% at genistein concentration below 75 μM (Figure 3A). And the same effect was observed in cytopathic effects (Figure 3B), viral protein expression (Figures 3C,D, ***P < 0.001), viral genome levels (Figure 3E, ***P < 0.001), plaque formation (Figures 3F,G, ***P < 0.001), and TCID50 (Figure 3H, ***P < 0.001). Therefore, genistein inhibited EV71 replication in HeLa cells.

### 3.2 Genistein inhibited the enteroviruses’ replication in host cells

**FIGURE 4:**
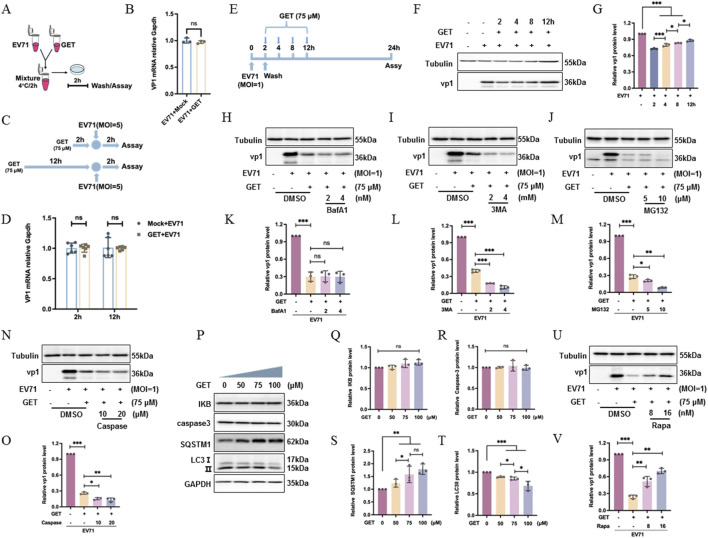
The mechanism of genistein in inhibiting EV71 replication in RD cells. **(A)** Diagram of the experimental workflow for pretreatment of EV71 with 75 μM of genistein (GET). **(B)** Following the workflow in **(A)**, relative viral mRNA level was detected at 2 h post-infection. Data were presented as the mean ± SD (N = 3) and analyzed with a T-test. **(C)** Diagram of the experimental workflow. **(D)** Following the workflow in **(C)** relative viral mRNA levels were detected. Data were presented as the mean ± SD (N = 3) and analyzed using a T-test. **(E)** Diagram of the experimental workflow. **(F)** According to the workflow of **(E)** Western blot analysis was done for VP1. **(G)** Corresponding to **(F)** the gray value ratio of the VP1 protein band to tubulin band is presented as the mean ± SD (N = 3) and analyzed by one-way ANOVA with Tukey’s multiple comparisons test. **(H)** Cells were infected with EV71 at an MOI of 1 for 2 h; at 2 h post-infection, GET with or without bafilomycin A1 (BafA1 2 nM or 4 nM) was applied; at 24 h post-infection, Western blot was done. **(I)** RD cells were infected with EV71 at an MOI of 1 for 2 h; at 2 h post-infection, GET with or without 3-methyladenine (3-MA 2 mM or 4 mM) was applied; at 24 h post-infection, Western blot was done. **(J)** RD cells were infected with EV71 at an MOI of 1 for 2 h; at 2 h post-infection, GET was administered; at 12 h post-infection, MG132 (5 μM and 10 μM) was added; at 24 h post-infection, Western blot was done. **(K–M)** Corresponding to **(H–J)** the gray value ratio of the VP1 protein band to tubulin band was presented as the mean ± SD (N = 3) and analyzed using one-way ANOVA with Tukey’s multiple comparisons test. **(N)** Cells were infected with EV71 at an MOI of 1 for 2 h; at 2 h post-infection, GET with or without caspase-3 inhibitor (10 μM and 20 μM) was applied; at 24 h post-infection, cells were collected for Western blot. **(O)** Corresponding to **(N)** the gray value ratio of the VP1 protein band to the Tubulin band was presented as the mean ± SD (N = 3) and analyzed using one-way ANOVA with Tukey’s multiple comparisons test. **(P)** Cells were treated with GET (0, 50, 75, and 100 μM); at 24 h post-treatment, cells were collected for Western blot analysis. **(Q–T)** Corresponding to **(P)** the gray value ratio of the targeted protein band to the control protein band was presented as the mean ± SD (N = 3) and analyzed using one-way ANOVA with Tukey’s multiple comparisons test. **(U)** Cells were infected with EV71 at an MOI of 1 for 2 h. At 2 h post-infection, cells were treated with GET for 14 h. At 16 h post-infection, cells were treated with rapamycin (Rapa, 8 nM and 16 nM) for 2 h, followed by treatment with 10% DMEM for 6 h. At 24 h post-infection, cells were collected for protein analysis. **(V)** Corresponding to **(U)** the gray value ratio of the target protein band to the tubulin band is presented as mean ± SD (N = 3) and analyzed using one-way ANOVA with Tukey’s multiple comparisons test. *P < 0.05, **P < 0.01, ***P < 0.001, ns: no significant difference. Tubulin or GAPDH was the loading control in Western blot.

The viral life cycle begins with the virus attaching to the surface of the host cell through the host cell receptors. This interaction enables the virus to enter the cell via receptor-mediated endocytosis, followed by viral genome replication, viral protein expression, viral assembly, and virion release (Solomon et al., 2010). To assess the effect of genistein on the viral life cycle, viral entry was analyzed by measuring viral genomic levels using real-time PCR analysis. First, 75 μM of genistein was mixed with EV71 for 2 h at 4 °C, then the medium containing both genistein and the virus was applied to RD cells for 2 h (Figure 4A). At 2 h post-infection, intracellular viral mRNA levels were measured by real-time PCR, revealing that genistein treatment did not affect viral entry (Figure 4B, *P* > 0.05), therefore, genistein does not directly target the virion to interfere with viral entry. To further investigate the effect of genistein on viral entry, RD cells were pretreated with 75 μM genistein for 2 h and 12 h to analyze whether genistein affects cell receptors, respectively. The 2-h treatment focuses on the short time effect of genistein on membrane receptors, while the 12-h treatment allows sufficient time for genistein acting on membrane receptors. After pretreatment, RD cells were infected with EV71 at MOI of 5 for 2 h at 37 °C. Then, at 2 h post-infection, intracellular viral mRNA levels were measured by real-time PCR (Figure 4C). Pretreatment with genistein for either 2 h or 12 h did not affect viral entry (Figure 4D, *P* > 0.05).

Then, RD cells were infected with EV71 at an MOI of 1 for 2 h. At 2 h post-infection, the virus-containing medium was discarded, and genistein was added at 2, 4, 8, and 12 h post-infection, respectively. Finally, at 24 h post-infection, RD cells were collected for Western blot analysis (Figure 4E). It was confirmed that the earlier the drug was administered, the stronger its inhibitory effect (Figures 4F,G, ***P < 0.001). These results indicated that genistein did not affect viral entry into host cells but inhibited viral replication within the host cells.

### 3.3 The antiviral mechanism of genistein

After confirming the anti-EV71 activity of genistein, its antiviral mechanism was analyzed. The autophagy pathway (Choi et al., 2018), proteasome (Choi et al., 2013), and caspases (Callus and Vaux, 2007) play important roles in viral replication; therefore, inhibitors of these pathways were used to observe their effects on genistein. Bafilomycin A1 and 3-MA were inhibitors of autophagy, MG132 was a proteasome inhibitor, and Z-VAD-FMK was a caspase inhibitor. First, RD cells were infected with EV71 at a MOI of 1 for 2 h. Two hours later, bafilomycin A1 (2 nM or 4 nM) was applied for 22 h. Then, cells were collected to assess viral protein expression. It was confirmed that genistein decreased viral protein expression (***P < 0.001), whereas bafilomycin A1 did not affect genistein’s action (*P* > 0.05) (Figures 4H,K). To further investigate the effect of autophagy inhibitor on genistein, RD cells were infected with EV71 at a MOI of 1 for 2 h. Two hours later, 3-MA (2 mM or 4 mM) was applied for 22 h. Cells were then collected for viral protein expression analysis. It was confirmed that genistein decreased viral protein expression (Figures 4I,L, ***P < 0.001), while 3-MA did not inhibit genistein’s effect; conversely, 3-MA enhanced genistein’s activity (Figures 4I,L, ***P < 0.001). Therefore, genistein likely does not activate autophagy to inhibit viral replication.

To analyze the effect of proteasome inhibitor on genistein, RD cells were infected with EV71 at a MOI of 1 for 2 h 10 h later, MG132 (5 μM or 10 μM) was administered for 12 h. Subsequently, cells were collected to assess viral protein expression. It was confirmed that genistein decreased viral protein expression (Figures 4J,M, ***P < 0.001), while MG132 further reduced the viral protein levels (Figures 4J,M, *P < 0.05). Therefore, genistein may not inhibit viral replication by activating the proteasome.

Then, RD cells were infected with EV71 at a MOI of 1 for 2 h. Two hours later, a caspase inhibitor (10 μM or 20 μM) was administered for 22 h. Cells were then collected for viral protein expression analysis. It was confirmed that genistein decreased viral protein expression (Figures 4N,O, ***P < 0.001), while the caspase inhibitor did not reverse genistein’s role, but further strength the role of genistein (Figures 4N,O, **P < 0.01). Thus, genistein likely does not inhibit viral replication by activating caspases.

Finally, to further clarify the roles of autophagy, proteasome, and caspase in genistein’s anti-EV71 activity, protein expression of these signaling pathways was examined. It was found that genistein did not alter the expression of the proteasome pathway marker IKB protein (which undergoes degradation in the proteasome) (Figures 4P,Q, *P* > 0.05), nor the caspase family protein caspase-3 (Figures 4P,R, *P* > 0.05). However, genistein increased the level of P62 (Figures 4P,S, **P < 0.01) and decreased the autophagy marker protein LC3II (Figures 4P,T, ***P < 0.001), indicating that genistein may inhibit autophagy and does not regulate the proteasome or caspase pathways.

Further confirming the role of autophagy in viral replication, RD cells were infected with EV71 at a MOI of 1 for 2 h. After 14 h, the autophagy activator rapamycin (8 nM or 16 nM) was applied for 8 h. Then, the cells were collected to assess viral protein expression. It was confirmed that genistein decreased viral protein expression (***P < 0.001), while rapamycin reversed the effect of genistein (Figures 4U,V, **P < 0.01). Therefore, genistein likely inhibits viral replication by suppressing autophagy.

### 3.4 Genistein inhibits autophagy

**FIGURE 5:**
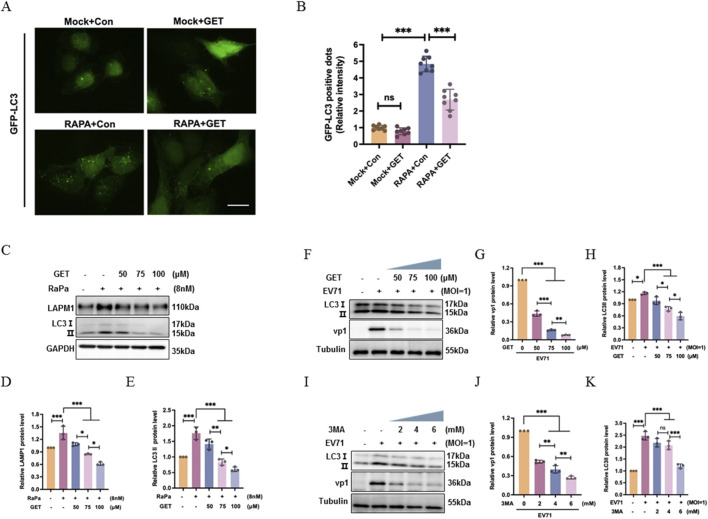
Genistein inhibited autophagy. **(A)** Cherry-GFP-LC3 plasmid was transfected into HeLa cells, at 24 h post transfection, HeLa cells treated with rapamycin (Rapa; 8 nM), genistein (GET; 75 μM), or both rapamycin (8 nM) and genistein (75 μM) for 24 h. Immunofluorescence analysis was recorded. Scale bars = 10 μm. **(B)** Corresponding to **(A)**, the GFP-LC3 positive dots was counted by ImageJ (N = 3, ***P < 0.001, ns: no significant difference). **(C)** RD cells were treated with rapamycin (8 nM) or together with doses of genistein (GET, 0, 50, 75, 100 μM) for 24 h. Then cells were collected for Western blotting analysis. **(D,E)** is calculated according to **(C)**. Data were presented as the mean ± SD (N = 3 independent experiments); Statistical analysis of was performed using one-way ANOVA with Tukey’s multiple comparisons test. (ns: No significant difference, *P < 0.05, **P < 0.01,***P < 0.001). **(F)** RD cells were infected with EV71 at a MOI of 1 for 2 h, at 2 h post infection, cells were treated with genistein (GET, 0, 50, 75, 100 μM) for 22 h. At 24 h post infection, cells were collected for Western blotting analysis. **(G,H)** is calculated according to **(F)**. The quantification of relative VP1 and LC3II/Tubulin were shown. Data were presented as the mean ± SD (N = 3 independent experiments); Statistical analysis of was performed using one-way ANOVA with Tukey’s multiple comparisons test. (*P < 0.05, **P < 0.01,***P < 0.001). **(I)** RD cells were infected with EV71 at a MOI of 1 for 2 h, at 2 h post infection, cells were treated with 3-MA (2, 4, 6 mM) for 22 h. At 24 h post infection, cells were collected for Western blotting analysis. **(J,K)** is calculated according to **(I)**. The quantification of relative VP1 and LC3II/Tubulin were shown. Data were presented as the mean ± SD (n = 3 independent experiments); Statistical analysis of was performed using one-way ANOVA with Tukey’s multiple comparisons test (*P < 0.05, **P < 0.01,***P < 0.001).

Rapamycin, as an autophagy activator, increased the number of GFP-LC3 puncta compared with the control treatment (***P < 0.001). Genistein reversed the effect of rapamycin (Figures 5A,B, ***P < 0.001). However, genistein did not decrease the baseline level of GFP-LC3 puncta relative to the control treatment (*P* > 0.05). To further confirm the genistein’s role in reversing rapamycin, RD cells were treated with rapamycin (8 nM) or together with doses of genistein (GET, 0, 50, 75, 100 μM) for 24 h. Then cells were collected for Western blotting analysis. Meanwhile it was noted that rapamycin upregulated the expression of LAMP1 and LC3, but genistein can reverse the role of rapamycin dose-dependently (Figures 5C–E, **P < 0.01, ***P < 0.001). Therefore, genistein can inhibit autophagy.

Then, the effect of genistein on virus-induced autophagy was investigated, RD cells were infected with EV71 at a MOI of 1 for 2 h, at 2 h post infection, cells were treated with genistein (GET, 0, 50, 75, 100 μM) for 22 h. At 24 h post infection, cells were collected for Western blotting analysis. It was found that EV71 upregulated LC3II (*P < 0.05), but genistein can reverse the role of EV71 to decrease the level of LC3II dose-dependently (Figures 5F,H, ***P < 0.001) and decrease the level of viral protein VP1 (Figures 5F,G, ***P < 0.001).

To further detect the role of autophagy in viral replication, autophagy inhibitor 3-MA was utilized to analyze the effect of autophagy on viral replication. RD cells were infected with EV71 at a MOI of 1 for 2 h, at 2 h post infection, cells were treated with 3-MA (2, 4, 6 mM) for 22 h. At 24 h post infection, cells were collected for Western blotting analysis. In antiviral role, autophagy inhibitor 3-MA has the same role as genistein in decreasing viral protein expression dose-dependently (Figures 5I,J, ***P < 0.001). EV71 infection increased the level of LC3II (Figures 5I,K, ***P < 0.001), while 3-MA decreased the level of LC3II (Figures 5I,K, ***P < 0.001). Therefore, genistein has the same ability as 3-MA in inhibiting autophagy and viral replication.

### 3.5 Genistein induced G2/M phase arrest

In viral replication, the host cell cycle plays a crucial role. For EV71, the virus induces and requires an arrest in the S phase, whereas synchronization at the G0/G1 phase and arrest at the G2/M phase inhibit viral replication (Yu et al., 2015).

**FIGURE 6:**
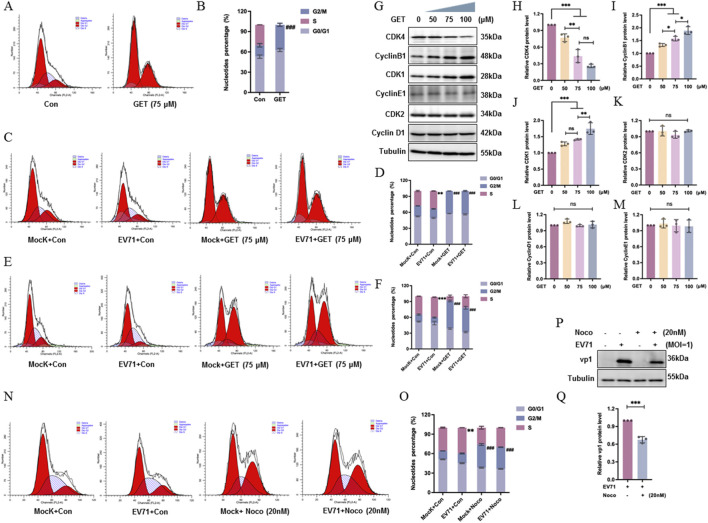
Genistein induces G2/M arrest, which inhibits viral replication. **(A,B)** HeLa cells were treated with genistein (GET, 75 μM) or 10% DMEM for 24 h. Flow cytometry was used to analyze the proportion of cells in G0/G1, G2/M, and S phases of the cell cycle. (G2/M of Con vs. GET, ###*P* < 0.001). **(C,D)** HeLa cells were infected with EV71 at a MOI of 5 for 2 h, then treated with genistein (75 μM) for 22 h. Flow cytometry analysis was performed to determine the distribution of cells in G0/G1, G2/M, and S phases. (S of Mock + Con vs. EV + Con, **P < 0.01; G2/M of Mock + Con vs. Mock + GET, ###*P* < 0.001; G2/M of EV + Con vs. EV + GET, ###*P* < 0.001). **(E,F)** RD cells were infected with EV71 at a MOI of 1 for 2 h, then treated with genistein (75 μM) for 22 h. Flow cytometry analysis was performed to determine the distribution of cells in G0/G1, G2/M, and S phases. (S of Mock + Con vs. EV + Con, ***P < 0.001; G2/M of Mock + Con vs. Mock + GET, ###*P* < 0.001; G2/M of EV + Con vs. EV + GET, ###*P* < 0.001) **(G–M)** HeLa cells were treated with genistein (0, 50, 75, 100 μM) for 24 h. Western blotting was used to analyze the relative protein levels of CDK4, CyclinB1, CDK1, CyclinE1, CDK2, and CyclinD1. Data were presented as mean ± SD (N = 3 independent experiments); statistical analysis was conducted using one-way ANOVA with Tukey’s multiple comparisons test. (ns: no significant difference; *P < 0.05; **P < 0.01; ***P < 0.001). **(N,O)** HeLa cells were treated with nocodazole (Noco; 20 nM) or 10% DMEM for 24 h. Flow cytometry was used to analyze the proportion of cells in G0/G1, G2/M, and S phases. (S of Mock + Con vs. EV + Con, **P < 0.01; G2/M of Mock + Con vs. Mock + Noco, ###*P* < 0.001; G2/M of EV + Con vs. EV + Noco, ###*P* < 0.001). **(P,Q)** HeLa cells were infected with EV71 at a MOI of 5 for 2 h; at 2 h post-infection, nocodazole (20 nM) was administered, and at 24 h post-infection, cells were collected for Western blotting. Tubulin served as the loading control. Data were presented as mean ± SEM (N = 3 independent experiments); statistical analysis was performed using a T-test. (***P < 0.001).

We also examined the effect of genistein on the cell cycle and found that compared to the control treatment, genistein increased the proportion of cells in the G2/M phase from (16.77 ± 2.54)% to (37.09 ± 2.38)% in HeLa cells (Figures 6A,B, ###*P* < 0.001), therefore, genistein induced G2/M arrest. In HeLa cells, EV71 induced S phase arrest from (27.96 ± 1.26)% to (33.63 ± 0.52)% compared to mock infection (Figures 6C,D, **P < 0.01), but genistein decreased this S phase arrest to 0%, corresponding with an increase in the G2/M phase in mock-infected cells (Figures 6C,D, ###*P* < 0.001). Meanwhile, in EV71 infected cells, genistein increased the ratio of G2/M from (16.78 ± 0.67)% to (43.72 ± 1.11)% (Figures 6C,D, ###*P* < 0.001).

Meanwhile, we investigated the effect of genistein on the cell cycle of RD cells, compared to the control treatment, genistein increased the proportion of cells in the G2/M phase from (13.30 ± 1.18)% to (52.53 ± 1.66)% in RD cells (Figures 6E,F, ###*P* < 0.001), therefore, genistein induced G2/M arrest. In RD cells, EV71 induced S phase arrest from (34.76 ± 0.38)% to (38.75 ± 0.47)% compared to mock infection (Figures 6E,F, ***P < 0.001), but genistein decreased this S phase arrest to (21.77 ± 2.76)%, corresponding with an increase in the G2/M phase in mock-infected cells (Figures 6E,F, ###*P* < 0.001). Meanwhile, in EV71 infected cells, genistein increased the ratio of G2/M from (10.50 ± 0.99)% to (46.14 ± 2.61)% (Figure 6E,F, ###*P* < 0.001) (Figures 6E,F, ###P < 0.001).

Additionally, genistein decreased the expression of CDK4 (Figures 6G,H, ***P < 0.001) and increased the expression of cyclin B1 (Figures 6G,I, ***P < 0.001) and CDK1 (Figures 6G,J, ***P < 0.001). Genistein did not affect the expression of cyclin E1, CDK2, or cyclin D1 (Figures 6G,K–M, *P* > 0.05), further confirming that genistein induces G2/M arrest. In this study, nocodazole induced G2/M phase arrest compared to control treatment (Figures 6N,O, ###*P* < 0.001), and when EV71 induced S phase arrest (Figures 6N,O, **P < 0.01), nocodazole increased the ratio of G2/M in EV71-infected cells (Figures 6N,O, ###*P* < 0.001) and inhibited viral protein expression (Figures 6P,Q, ***P < 0.001). Therefore, drug-induced G2/M arrest inhibits viral replication.

### 3.6 Genistein inhibited the EV68 and CA6 replication

**FIGURE 7:**
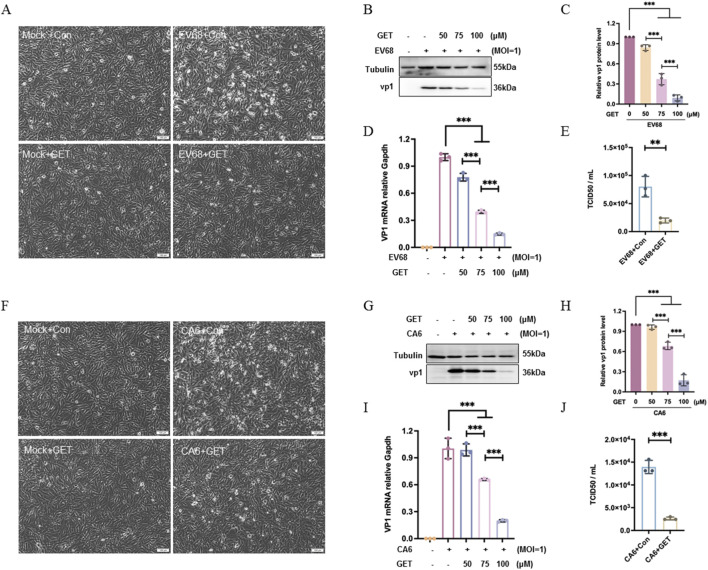
Genistein inhibits EV68 and CA6 replication in RD cells. RD cells were infected with EV68 or CA6 at an MOI of 1 for 2 h. At 2 h post-infection, cells were treated with 10% DMEM or genistein (75 μM GET, or various doses of GET). At 24 h post-infection, cells were collected for analysis. **(A,F)** Observation of round and floating cells using an inverted microscope. N = 3 independent experiments. **(B,G)** Western blot analysis of VP1 expression in RD cells infected with EV68 **(B)** or CA6 **(G)**. Tubulin served as the loading control. **(C,H)** Corresponding to **(B,G)** respectively. The gray values of the VP1 protein bands normalized to Tubulin bands were presented as mean ± SD (N = 3 independent experiments). Statistical analysis was performed using one-way ANOVA with Tukey’s multiple comparisons test (ns: no significant difference; *P < 0.05; **P < 0.01; ***P < 0.001). **(D,I)** Relative mRNA levels of the viral genome in RD cells infected with EV68 **(D)** or CA6 **(I)**. Data were shown as mean ± SD (N = 3 independent experiments); statistical analysis was done by one-way ANOVA with Tukey’s multiple comparisons test (ns: no significant difference; ***P < 0.001). **(E,J)** Intracellular and supernatant progeny virions were titrated using RD cells by TCID50/ml assay. Data are presented as mean ± SD (N = 3 independent experiments); statistical analysis was performed using T-test (**P < 0.01; ***P < 0.001).

To further confirm that the antiviral ability of genistein is not limited to EV71, its effect on EV68 and CA6 was investigated. Human EV68, an enterovirus, was first isolated in 1962 in California. Clinical symptoms of EV68 infection include respiratory illness, with complications that may require hospitalization and can even be fatal (Su et al., 2022). The coxsackievirus A6 (CA6) strain has been associated with severe global outbreaks since 2008. CA6 is linked to a more severe and extensive form of HFMD, affecting both children and adults (Ramirez-Fort et al., 2014). RD cells were infected with EV68 and CA6 at MOI of 1 for 2 h respectively, followed by treatment with 75 μM of genistein for 22 h. It was confirmed that EV68 induced cytopathic effects in RD cells, which genistein reversed (Figure 7A). Genistein was found to dose-dependently reduce viral protein expression (Figures 7B,C, ***P < 0.001) and viral mRNA levels (Figure 7D, ***P < 0.001), and it decreased the number of virions (Figure 7E, **P < 0.01). Similarly, CA6 induced cytopathic effects in RD cells, which genistein inhibited (Figure 7F). Genistein also dose-dependently reduced viral protein expression (Figures 7G,H, ***P < 0.001) and viral mRNA levels (Figure 7I, ***P < 0.001), and decreased the number of virions (Figure 7J, ***P < 0.001). Therefore, genistein also inhibited the replication of other enteroviruses EV68 and CA6, it has broad-spectrum antiviral effects for enteroviruses.

### 3.7 Genistein protected neonatal mouse from the damage of EV71

**FIGURE 8:**
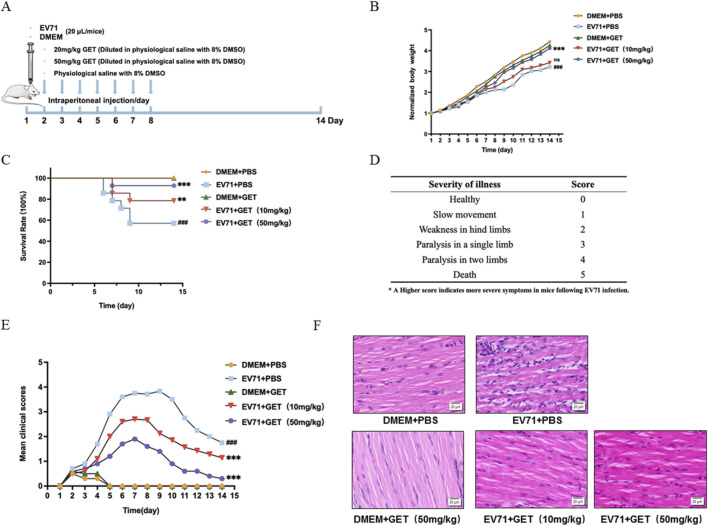
Genistein protects neonatal mice from EV71-induced damage. **(A)** Experimental workflow diagram for mice injected with EV71 and genistein. Newborn ICR mice were intracranially inoculated with EV71 (10^7.0^ CCID50/ml, 20 μL) or DMEM (20 μL) within 24 h of birth, followed by intraperitoneal injections of genistein (10 mg/kg, 50 mg/kg) or PBS (8% DMSO-PBS) for 7 consecutive days. From day 1 to day 14 after birth, body weight, mortality, and illness severity were recorded. **(B)** Effects of genistein (10 mg/kg, 50 mg/kg) on body weight of newborn ICR mice challenged with EV71 (CC077). N = 10. A two-way ANOVA was performed to compare differences between test groups and the virus control (EV71+PBS vs. DMEM + PBS, ###*P* < 0.001; EV71 + 10 mg/kg GET vs. EV71+PBS, ns: no significant difference; EV71 + 50 mg/kg GET vs. EV71+PBS, ***P < 0.001). **(C)** Effects of genistein (10 mg/kg, 50 mg/kg) on survival rates of newborn mice challenged with EV71 (CC077). Log-rank (Mantel-Cox) test was performed (EV71+PBS vs. DMEM + PBS, ###*P* < 0.001; EV71 + 10 mg/kg GET vs. EV71+PBS, **P < 0.01; EV71 + 50 mg/kg GET vs. EV71+PBS, ***P < 0.001). **(D)** Criteria for rating illness severity in mice. **(E)** Effects of genistein (10 mg/kg, 50 mg/kg) on clinical scores of newborn mice challenged with EV71. A one-way ANOVA was performed to compare differences between test groups and virus control (EV71+PBS vs. DMEM + PBS, ###*P* < 0.001; EV71 + 10 mg/kg GET vs. EV71+PBS, ***P < 0.001; EV71 + 50 mg/kg GET vs. EV71+PBS, ***P < 0.001). **(F)** Effects of genistein (10 mg/kg, 50 mg/kg) on histopathological analysis of newborn mice challenged with EV71. N = 3 replicates in each concentration.

To further demonstrate the role of genistein in EV71 replication, an *in vivo* experiment was conducted (Figure 8A). For the body weights of the newborn mice, EV71 injection (EV71+PBS) caused a decrease compared to the DMEM + PBS group (###*P* < 0.001). Administration of 10 mg/kg genistein (EV71 + 10 mg/kg GET) did not restore the body weight of EV71-inoculated mice (EV71+PBS) (*P* > 0.05), but 50 mg/kg genistein (EV71 + 50 mg/kg GET) significantly increased the body weight of EV71-inoculated mice (Figure 8B, ***P < 0.001).

Regarding survival rates of neonatal mice, EV71 injection (EV71+PBS) resulted in 57.14% survival rate compared to the 100% survival rate in the DMEM + PBS group (###*P* < 0.001). Treatment with 10 mg/kg genistein (EV71 + 10 mg/kg GET) improved survival rates to 78.57% in EV71-infected mice (EV71+PBS) (**P < 0.01), and 50 mg/kg genistein (EV71 + 50 mg/kg GET) further increased survival rates to 92.85% in EV71-inoculated mice (Figure 8C, ***P < 0.001).

The severity of illness in mice was scored, with higher scores indicating greater damage (Pei et al., 2022) (Figure 8D). Compared to the control group (DMEM + PBS), EV71 injection (EV71+PBS) caused significantly higher scores (###*P* < 0.001). Both 10 mg/kg genistein (EV71 + 10 mg/kg GET) and 50 mg/kg genistein (EV71 + 50 mg/kg GET) significantly reduced illness scores in EV71-infected mice (Figure 8E, ***P < 0.001). For pathological changes, the virus-treated group showed more lymphocyte infiltration and muscle fiber rupture, but 10 mg/kg and 50 mg/kg the genistein-treated group could weaken these pathological changes (Figure 8F). Therefore, genistein demonstrates protective effects against EV71-induced damage *in vivo*.

## 4 Discussion

Genistein exerts a direct inhibitory effect on the viral life cycle. It has been shown to block viral entry in multiple systems (Nicola et al., 2005; Stantchev et al., 2007; Guo et al., 2012; Zhang et al., 2018; Li et al., 2020; Synowiec et al., 2023). Beyond entry, genistein significantly reduces both viral gene transcription after infection and virion release (Qian et al., 2014). Mechanistically, genistein interacts with the DNA-dependent RNA polymerase subunit RPO30 to inhibit Orf virus replication (Lv et al., 2024). It also inhibits African swine fever virus replication *in vitro* by blocking the synthesis of ASFV type II topoisomerase (Arabyan et al., 2018). Genistein derivatives have been identified as potential inhibitors of the SARS-CoV-2 main protease (Liu et al., 2022). In the present study, genistein did not affect viral entry but inhibited genome replication, viral protein expression, and virion production, suggesting interference with multiple stages of the viral life cycle.

Genistein is a general protein kinase inhibitor with selectivity toward tyrosine residues. It interferes with caveola-mediated endocytosis by inhibiting viral internalization through caveolae; biochemically, it blocks the phosphorylation of tyrosine kinase involved in caveosome formation. The viral capsid proteins VP1 and VP2 have binding sites for the main receptor hSCARB-2 (human scavenger receptor class B, member 2), allowing EV71 entry into cells via clathrin-mediated endocytosis (Zhou et al., 2019), a classical pathway for most viruses to enter host cells (Zhang et al., 2018). In contrast, in the absence of hSCARB-2, other receptors or co-receptors such as PSGL-1 (P-selectin glycoprotein ligand 1), sialylated glycans, annexin 2, heparan sulfate, heat shock protein 90, cyclophilin A, vimentin, nucleolin, fibronectin, prohibitin, and human tryptophanyl-tRNA synthetase attach to the virus and initiate caveolae-mediated or endophilin-A2-mediated endocytosis (Lin et al., 2013; Hu et al., 2023). In RD cells, which have high levels of hSCARB-2, EV71 binds to hSCARB-2 and enters host cells through clathrin-mediated endocytosis (Hussain et al., 2011). Therefore, in RD cells, genistein, as an inhibitor of caveolae-mediated endocytosis, cannot block EV71 entry. However, in cells lacking the hSCARB-2 receptor, genistein may have the ability to prevent viral entry.

Genistein inhibits the cyclic GMP-AMP (cGAMP) synthase (cGAS-STING) pathway at the level of cGAMP transfer and its sensing by STING to support viral replication (Ullah et al., 2022). By pretreating macrophages with genistein, the level of IL-8 mRNA induced by virus treatment is severely reduced (Legastelois et al., 1998). The New World arenavirus Pichindé-induced phosphorylation of the activating transcription factor-2 protein (ATF-2) and cyclic adenosine monophosphate response element-binding protein (CREB) is inhibited following genistein treatment (Vela et al., 2008). Genistein also inhibits the interaction between lens epithelium-derived growth factor and HIV integrase to block HIV replication (Yin et al., 2022). However, there has been no report on genistein regulating autophagy to inhibit EV71 replication. The interaction between autophagy and viruses can explain autophagy’s role in viral infection, which can have either proviral or antiviral functions. On one hand, EV71 induces autophagy to promote its own replication (Huang et al., 2009; Lee et al., 2014; Chen et al., 2018; Li et al., 2019; Wang et al., 2019; Wang et al., 2024; Tian et al., 2025; You et al., 2025), and on the other hand, autophagy inhibits EV71 replication through degrading virus particles or components upon viral infection (Wang et al., 2022). Viruses can induce autophagy but then hijack autophagosomes as replication sites or hijack the secretory autophagy pathway to promote the maturation and release of virus particles, thereby increasing replication and transmission efficiency. Genistein has been reported to decrease autophagy in some studies (Mohan et al., 2013; Nazim and Park, 2015; Wu et al., 2023; Wu et al., 2024), while other studies report it increases autophagy (Lee et al., 2016; Wang et al., 2018; Pierzynowska et al., 2019; Zhang et al., 2019; Pierzynowska et al., 2024). In this study, genistein is confirmed to decrease the autophagy marker LC3II expression and aggregation and simultaneously decrease the lysosome marker LAMP1 expression, as well as inhibit rapamycin’s ability to upregulate autophagy. Therefore, our study supports that genistein decreases autophagy. Class III PI3Ks are responsible for autophagosome formation and autophagy flux. 3-Methyladenine is a widely used autophagy inhibitor through its inhibitory effect on class III PI3K (Safaroghli-Azar et al., 2023). Genistein has the same role as 3-methyladenine in inhibiting EV71 replication; this anti-EV71 effect can be reversed by rapamycin. Thus, genistein likely inhibits autophagy similarly to 3-MA by acting at the initiation phase of autophagy, which provides a replication site for viral replication. Our study further confirms that autophagy, especially the initiation process, promotes viral replication.

Regarding the regulation of genistein on the cell cycle, numerous reports exist, most of which confirm that genistein arrests the cell cycle at the G2/M phase (Ismail et al., 2007; Zhang et al., 2013; Cui et al., 2014), consistent with our study. It has been reported that genistein-induced cell cycle arrest in G2 promotes HIV-1 replication because HIV-1 induces cell cycle arrest in G2/M to enhance viral replication (Gozlan et al., 1998). However, whether cell cycle arrest induced by genistein affects EV71 replication remains unclear. This study confirms that G2/M arrest inhibits EV71 replication, and genistein induces G2/M arrest. Therefore, genistein induces G2/M arrest to inhibit viral replication, and altering the cell cycle state can intervene in viral replication.

More importantly, in neonatal mice, genistein protects mice from damage caused by EV71 infection, as evidenced by improvements in body weight, survival rate, and general condition; genistein is also safe for neonatal mice. Meanwhile, genistein also inhibits EV68 and CA6 replication, genistein has broad-spectrum antiviral effects for enteroviruses.

## 5 Conclusion

Genistein protects the host from EV71-induced damage both *in vivo and in vitro.* Genistein inhibits EV71 replication by inhibiting autophagy and causing accumulation of cells in the G2/M phase. Genistein is a promising candidate drug for combating enterovirus.

## Data Availability

The raw data supporting the conclusions of this article will be made available by the authors, without undue reservation.

## References

[B1] Andres A. Donovan S. M. Kuhlenschmidt M. S. (2009). Soy isoflavones and virus infections. J. Nutr. Biochem. 20, 563–569. 10.1016/j.jnutbio.2009.04.004 19596314 PMC7125569

[B2] Arabyan E. Hakobyan A. Kotsinyan A. Karalyan Z. Arakelov V. Arakelov G. (2018). Genistein inhibits African swine fever virus replication *in vitro* by disrupting viral DNA synthesis. Antivir. Res. 156, 128–137. 10.1016/j.antiviral.2018.06.014 29940214 PMC7127377

[B3] Bagga S. Bouchard M. J. (2014). Cell cycle regulation during viral infection. Methods Mol. Biol. 1170, 165–227. 10.1007/978-1-4939-0888-2_10 24906315 PMC7122065

[B4] Callus B. A. Vaux D. L. (2007). Caspase inhibitors: viral, cellular and chemical. Cell Death Differ. 14, 73–78. 10.1038/sj.cdd.4402034 16946729

[B5] Chen D. Feng C. Tian X. Zheng N. Wu Z. (2018). Promyelocytic leukemia restricts enterovirus 71 replication by inhibiting autophagy. Front. Immunol. 9, 1268. 10.3389/fimmu.2018.01268 29922292 PMC5996053

[B6] Choi A. G. Wong J. Marchant D. Luo H. (2013). The ubiquitin-proteasome system in positive-strand RNA virus infection. Rev. Med. Virol. 23, 85–96. 10.1002/rmv.1725 22782620 PMC7169083

[B7] Choi Y. Bowman J. W. Jung J. U. (2018). Autophagy during viral infection - a double-edged sword. Nat. Rev. Microbiol. 16, 341–354. 10.1038/s41579-018-0003-6 29556036 PMC6907743

[B8] Cui S. Wienhoefer N. Bilitewski U. (2014). Genistein induces morphology change and G2/M cell cycle arrest by inducing p38 MAPK activation in macrophages. Int. Immunopharmacol. 18, 142–150. 10.1016/j.intimp.2013.11.016 24290959

[B9] Deretic V. Saitoh T. Akira S. (2013). Autophagy in infection, inflammation and immunity. Nat. Rev. Immunol. 13, 722–737. 10.1038/nri3532 24064518 PMC5340150

[B10] Fuloria S. Yusri M. a.A. Sekar M. Gan S. H. Rani N. Lum P. T. (2022). Genistein: a potential natural lead molecule for new drug design and development for treating memory impairment. Molecules 27, 265. 10.3390/molecules27010265 35011497 PMC8746870

[B11] Gozlan J. Lathey J. L. Spector S. A. (1998). Human immunodeficiency virus type 1 induction mediated by genistein is linked to cell cycle arrest in G2. J. Virol. 72, 8174–8180. 10.1128/JVI.72.10.8174-8180.1998 9733859 PMC110162

[B12] Guo C. J. Wu Y. Y. Yang L. S. Yang X. B. He J. Mi S. (2012). Infectious spleen and kidney necrosis virus (A fish iridovirus) enters mandarin fish fry cells *via* caveola-dependent endocytosis. J. Virol. 86, 2621–2631. 10.1128/JVI.06947-11 22171272 PMC3302275

[B13] Hu Y. Musharrafieh R. Zheng M. Wang J. (2020). Enterovirus D68 antivirals: past, present, and future. ACS Infect. Dis. 6, 1572–1586. 10.1021/acsinfecdis.0c00120 32352280 PMC8055446

[B14] Hu K. Onintsoa Diarimalala R. Yao C. Li H. Wei Y. (2023). EV-A71 mechanism of entry: Receptors/co-receptors, related pathways and inhibitors. Viruses 15, 785. 10.3390/v15030785 36992493 PMC10051052

[B15] Huang S. C. Chang C. L. Wang P. S. Tsai Y. Liu H. S. (2009). Enterovirus 71-induced autophagy detected *in vitro* and *in vivo* promotes viral replication. J. Med. Virol. 81, 1241–1252. 10.1002/jmv.21502 19475621 PMC7166624

[B16] Hussain K. M. Leong K. L. Ng M. M. Chu J. J. (2011). The essential role of clathrin-mediated endocytosis in the infectious entry of human enterovirus 71. J. Biol. Chem. 286, 309–321. 10.1074/jbc.M110.168468 20956521 PMC3012988

[B17] Ismail I. A. Kang K. S. Lee H. A. Kim J. W. Sohn Y. K. (2007). Genistein-induced neuronal apoptosis and G2/M cell cycle arrest is associated with MDC1 up-regulation and PLK1 down-regulation. Eur. J. Pharmacol. 575, 12–20. 10.1016/j.ejphar.2007.07.039 17706963

[B18] Jin P. Li J. Zhang X. Meng F. Zhou Y. Yao X. (2016). Validation and evaluation of serological correlates of protection for inactivated enterovirus 71 vaccine in children aged 6-35 months. Hum. Vaccin Immunother. 12, 916–921. 10.1080/21645515.2015.1118595 26751765 PMC4962943

[B19] Křížová L. Dadáková K. Kašparovská J. Kašparovský T. (2019). Isoflavones. Molecules 24, 1076. 10.3390/molecules24061076 30893792 PMC6470817

[B20] Lee Y. R. Wang P. S. Wang J. R. Liu H. S. (2014). Enterovirus 71-induced autophagy increases viral replication and pathogenesis in a suckling mouse model. J. Biomed. Sci. 21, 80. 10.1186/s12929-014-0080-4 25139436 PMC4237791

[B21] Lee K. Y. Kim J. R. Choi H. C. (2016). Genistein-induced LKB1-AMPK activation inhibits senescence of VSMC through autophagy induction. Vasc. Pharmacol. 81, 75–82. 10.1016/j.vph.2016.02.007 26924458

[B22] Legastelois I. Levrey H. Greenland T. Mornex J. F. Cordier G. (1998). Visna-maedi virus induces interleukin-8 in sheep alveolar macrophages through a tyrosine-kinase signaling pathway. Am. J. Respir. Cell Mol. Biol. 18, 532–537. 10.1165/ajrcmb.18.4.2812 9533941

[B23] Li P. Yang S. Hu D. Wei D. Lu J. Zheng H. (2019). Enterovirus 71 VP1 promotes mouse schwann cell autophagy *via* ER stress-mediated PMP22 upregulation. Int. J. Mol. Med. 44, 759–767. 10.3892/ijmm.2019.4218 31173167

[B24] Li M. Yan P. Liu Z. Cai D. Luo Y. Wu X. (2020). Muscovy duck reovirus enters susceptible cells *via* a caveolae-mediated endocytosis-like pathway. Virus Res. 276, 197806. 10.1016/j.virusres.2019.197806 31704247

[B25] Lin H. Y. Yang Y. T. Yu S. L. Hsiao K. N. Liu C. C. Sia C. (2013). Caveolar endocytosis is required for human PSGL-1-mediated enterovirus 71 infection. J. Virol. 87, 9064–9076. 10.1128/JVI.00573-13 23760234 PMC3754029

[B26] Liu J. Zhang L. Gao J. Zhang B. Liu X. Yang N. (2022). Discovery of genistein derivatives as potential SARS-CoV-2 main protease inhibitors by virtual screening, molecular dynamics simulations and ADMET analysis. Front. Pharmacol. 13, 961154. 10.3389/fphar.2022.961154 36091808 PMC9452787

[B27] Lv P. Fang Z. Guan J. Lv L. Xu M. Liu X. (2024). Genistein is effective in inhibiting orf virus infection *in vitro* by targeting viral RNA polymerase subunit RPO30 protein. Front. Microbiol. 15, 1336490. 10.3389/fmicb.2024.1336490 38389526 PMC10882098

[B28] Martin J. H. Crotty S. Warren P. Nelson P. N. (2007). Does an Apple a day keep the doctor away because a phytoestrogen a day keeps the virus at bay? A review of the anti-viral properties of phytoestrogens. Phytochemistry 68, 266–274. 10.1016/j.phytochem.2006.11.018 17182070

[B29] Mathew S. Vazhappilly C. G. (2020). Recent pharmacological advances on genistein in clinical trials. Excli J. 19, 1120–1123. 10.17179/excli2020-2675 33088249 PMC7573176

[B30] Middleton E. Jr. Kandaswami C. Theoharides T. C. (2000). The effects of plant flavonoids on mammalian cells: implications for inflammation, heart disease, and cancer. Pharmacol. Rev. 52, 673–751. 10.1016/S0031-6997(24)01472-8 11121513

[B31] Mohan N. Chakrabarti M. Banik N. L. Ray S. K. (2013). Combination of LC3 shRNA plasmid transfection and genistein treatment inhibited autophagy and increased apoptosis in malignant neuroblastoma in cell culture and animal models. PLoS One 8, e78958. 10.1371/journal.pone.0078958 24205354 PMC3800129

[B32] Nascimento R. Costa H. Parkhouse R. M. (2012). Virus manipulation of cell cycle. Protoplasma 249, 519–528. 10.1007/s00709-011-0327-9 21986922

[B33] Nazim U. M. Park S. Y. (2015). Genistein enhances TRAIL-induced cancer cell death *via* inactivation of autophagic flux. Oncol. Rep. 34, 2692–2698. 10.3892/or.2015.4247 26352862

[B34] Ng Q. He F. Kwang J. (2015). Recent progress towards novel EV71 anti-therapeutics and vaccines. Viruses 7, 6441–6457. 10.3390/v7122949 26670245 PMC4690872

[B35] Nicola A. V. Hou J. Major E. O. Straus S. E. (2005). Herpes simplex virus type 1 enters human epidermal keratinocytes, but not neurons, *via* a pH-dependent endocytic pathway. J. Virol. 79, 7609–7616. 10.1128/JVI.79.12.7609-7616.2005 15919913 PMC1143659

[B36] Pei Z. Wang H. Zhao Z. Chen X. Huan C. Zhang W. (2022). Chemokine PF4 Inhibits EV71 and CA16 infections at the entry stage. J. Virol. 96, e0043522. 10.1128/jvi.00435-22 35579435 PMC9175630

[B37] Pierzynowska K. Podlacha M. Gaffke L. Majkutewicz I. Mantej J. Węgrzyn A. (2019). Autophagy-dependent mechanism of genistein-mediated elimination of behavioral and biochemical defects in the rat model of sporadic alzheimer's disease. Neuropharmacology 148, 332–346. 10.1016/j.neuropharm.2019.01.030 30710571

[B38] Pierzynowska K. Podlacha M. Gaffke L. Rintz E. Wiśniewska K. Cyske Z. (2024). Correction of symptoms of huntington disease by genistein through FOXO3-mediated autophagy stimulation. Autophagy 20, 1159–1182. 10.1080/15548627.2023.2286116 37992314 PMC11135876

[B39] Qian K. Gao A. J. Zhu M. Y. Shao H. X. Jin W. J. Ye J. Q. (2014). Genistein inhibits the replication of Avian leucosis virus subgroup J in DF-1 cells. Virus Res. 192, 114–120. 10.1016/j.virusres.2014.08.016 25197039

[B40] Ramirez-Fort M. K. Downing C. Doan H. Q. Benoist F. Oberste M. S. Khan F. (2014). Coxsackievirus A6 associated hand, foot and mouth disease in adults: clinical presentation and review of the literature. J. Clin. Virol. 60, 381–386. 10.1016/j.jcv.2014.04.023 24932735

[B41] Rimbach G. Boesch-Saadatmandi C. Frank J. Fuchs D. Wenzel U. Daniel H. (2008). Dietary isoflavones in the prevention of cardiovascular disease--a molecular perspective. Food Chem. Toxicol. 46, 1308–1319. 10.1016/j.fct.2007.06.029 17689850

[B42] Rizzo G. Baroni L. (2018). Soy, soy foods and their role in vegetarian diets. Nutrients 10, 43. 10.3390/nu10010043 29304010 PMC5793271

[B43] Safaroghli-Azar A. Sanaei M. J. Pourbagheri-Sigaroodi A. Bashash D. (2023). Phosphoinositide 3-kinase (PI3K) classes: from cell signaling to endocytic recycling and autophagy. Eur. J. Pharmacol. 953, 175827. 10.1016/j.ejphar.2023.175827 37269974

[B44] Solomon T. Lewthwaite P. Perera D. Cardosa M. J. Mcminn P. Ooi M. H. (2010). Virology, epidemiology, pathogenesis, and control of enterovirus 71. Lancet Infect. Dis. 10, 778–790. 10.1016/S1473-3099(10)70194-8 20961813

[B45] Stantchev T. S. Markovic I. Telford W. G. Clouse K. A. Broder C. C. (2007). The tyrosine kinase inhibitor genistein blocks HIV-1 infection in primary human macrophages. Virus Res. 123, 178–189. 10.1016/j.virusres.2006.09.004 17030448 PMC1847631

[B46] Su Y. Wu T. Yu X. Y. Huo W. B. Wang S. H. Huan C. (2022). Inhibitory effect of tanshinone IIA, resveratrol and silibinin on enterovirus 68 production through inhibiting ATM and DNA-PK pathway. Phytomedicine 99, 153977. 10.1016/j.phymed.2022.153977 35305353

[B47] Synowiec A. Dąbrowska A. Pachota M. Baouche M. Owczarek K. Niżański W. (2023). Feline herpesvirus 1 (FHV-1) enters the cell by receptor-mediated endocytosis. J. Virol. 97, e0068123. 10.1128/jvi.00681-23 37493545 PMC10506464

[B48] Tian X. Yuan M. Li L. Chen D. Liu B. Zou X. (2025). Enterovirus 71 induces mitophagy *via* PINK1/Parkin signaling pathway to promote viral replication. Faseb J. 39, e70659. 10.1096/fj.202403315R 40396408

[B49] Ullah T. R. Balka K. R. Ambrose R. L. Pépin G. Wilce M. C. J. Wilce J. A. (2022). Genistein targets STING-driven antiviral responses. mBio 13, e0206422. 10.1128/mbio.02064-22 35924852 PMC9426420

[B50] Vela E. M. Bowick G. C. Herzog N. K. Aronson J. F. (2008). Genistein treatment of cells inhibits arenavirus infection. Antivir. Res. 77, 153–156. 10.1016/j.antiviral.2007.09.005 17961732 PMC2259390

[B51] Vincent A. Fitzpatrick L. A. (2000). Soy isoflavones: are they useful in menopause? Mayo Clin. Proc. 75, 1174–1184. 10.4065/75.11.1174 11075748

[B52] Wang Y. Li Y. Zhang T. Chi Y. Liu M. Liu Y. (2018). Genistein and Myd88 activate autophagy in high glucose-induced renal podocytes *in vitro* . Med. Sci. Monit. 24, 4823–4831. 10.12659/MSM.910868 29999001 PMC6069420

[B53] Wang H. Guo T. Yang Y. Yu L. Pan X. Li Y. (2019). Lycorine derivative LY-55 inhibits EV71 and CVA16 replication through downregulating autophagy. Front. Cell Infect. Microbiol. 9, 277. 10.3389/fcimb.2019.00277 31448243 PMC6692562

[B54] Wang S. Qiao J. Chen Y. Tian L. Sun X. (2022). Urolithin A inhibits enterovirus 71 replication and promotes autophagy and apoptosis of infected cells *in vitro* . Arch. Virol. 167, 1989–1997. 10.1007/s00705-022-05471-1 35790643

[B55] Wang S. Liu R. Zhou Y. Xu J. Su A. Zheng D. (2024). TUDCA inhibits EV71 replication by regulating ER stress signaling pathway and suppressing autophagy. Diagn Microbiol. Infect. Dis. 110, 116500. 10.1016/j.diagmicrobio.2024.116500 39213902

[B56] Wu G. Song D. Wu H. Zhao F. Ding W. Wang Z. (2023). Genistein ameliorates starvation-induced porcine follicular granulosa cell apoptosis. Reproduction 166, 451–458. 10.1530/REP-23-0156 37855439

[B57] Wu J. Feng A. Liu C. Zhou W. Li K. Liu Y. (2024). Genistein alleviates doxorubicin-induced cardiomyocyte autophagy and apoptosis *via* ERK/STAT3/c-Myc signaling pathway in rat model. Phytother. Res. 38, 3921–3934. 10.1002/ptr.8236 38818771

[B58] Yin Z. H. Yan H. L. Pan Y. Zhang D. W. Yan X. (2022). Evaluation of a flavonoid library for inhibition of interaction of HIV-1 integrase with human LEDGF/p75 towards a structure-activity relationship. Ann. Med. 54, 1590–1600. 10.1080/07853890.2022.2081869 35658757 PMC9176681

[B59] You Q. Wu J. Lyu R. Cai Y. Jiang N. Liu Y. (2025). 6-thioguanine inhibits EV71 replication by reducing BIRC3-mediated autophagy. BMC Microbiol. 25, 53. 10.1186/s12866-025-03752-8 39881250 PMC11776205

[B60] Yu J. Zhang L. Ren P. Zhong T. Li Z. Wang Z. (2015). Enterovirus 71 mediates cell cycle arrest in S phase through non-structural protein 3D. Cell Cycle 14, 425–436. 10.4161/15384101.2014.980631 25659038 PMC4353240

[B61] Zhang Z. Wang C. Z. Du G. J. Qi L. W. Calway T. He T. C. (2013). Genistein induces G2/M cell cycle arrest and apoptosis *via* ATM/p53-dependent pathway in human colon cancer cells. Int. J. Oncol. 43, 289–296. 10.3892/ijo.2013.1946 23686257 PMC3742162

[B62] Zhang F. Guo H. Zhang J. Chen Q. Fang Q. (2018). Identification of the caveolae/raft-mediated endocytosis as the primary entry pathway for aquareovirus. Virology 513, 195–207. 10.1016/j.virol.2017.09.019 29102889

[B63] Zhang H. Yang X. Pang X. Zhao Z. Yu H. Zhou H. (2019). Genistein protects against ox-LDL-induced senescence through enhancing SIRT1/LKB1/AMPK-mediated autophagy flux in HUVECs. Mol. Cell Biochem. 455, 127–134. 10.1007/s11010-018-3476-8 30443855

[B64] Zhou D. Zhao Y. Kotecha A. Fry E. E. Kelly J. T. Wang X. (2019). Unexpected mode of engagement between enterovirus 71 and its receptor SCARB2. Nat. Microbiol. 4, 414–419. 10.1038/s41564-018-0319-z 30531980

